# Mutations in the *NUP93*, *NUP107* and *NUP160* genes cause steroid-resistant nephrotic syndrome in Chinese children

**DOI:** 10.1186/s13052-024-01656-3

**Published:** 2024-04-22

**Authors:** Yanxinli Han, Hongyu Sha, Yuan Yang, Zhuowei Yu, Lanqi Zhou, Yi Wang, Fengjie Yang, Liru Qiu, Yu Zhang, Jianhua Zhou

**Affiliations:** 1 Department of Pediatrics, Tongji Hospital, Tongji Medical College, Huazhong University of Science & Technology, Wuhan, Hubei province 430030 China; 2 Department of Pharmacy, Yantai Yuhuangding Hospital, Yantai, Shandong Province 264000 China

**Keywords:** End-stage kidney disease, *NUP93*, *NUP107*, *NUP160*, Steroid-resistant nephrotic syndrome, Gene variants

## Abstract

**Background:**

The variants of nucleoporins are extremely rare in hereditary steroid-resistant nephrotic syndrome (SRNS). Most of the patients carrying such variants progress to end stage kidney disease (ESKD) in their childhood. More clinical and genetic data from these patients are needed to characterize their genotype–phenotype relationships and elucidate the role of nucleoporins in SRNS.

**Methods:**

Four patients of SRNS carrying biallelic variants in the *NUP93*, *NUP107* and *NUP160* genes were presented. The clinical and molecular genetic characteristics of these patients were summarized, and relevant literature was reviewed.

**Results:**

All four patients in this study were female and initially presented with SRNS. The median age at the onset of the disease was 5.08 years, ranging from 1 to 10.5 years. Among the four patients, three progressed to ESKD at a median age of 7 years, ranging from 1.5 to 10.5 years, while one patient reached stage 3 chronic kidney disease (CKD3). Kidney biopsies revealed focal segmental glomerulosclerosis in three patients. Biallelic variants were detected in *NUP93* in one patient, *NUP107* in two patients, as well as *NUP160* in one patient respectively. Among these variants, five yielded single amino acid substitutions, one led to nonsense mutation causing premature termination of NUP107 translation, one caused a single nucleotide deletion resulting in frameshift and truncation of NUP107. Furthermore, one splicing donor mutation was observed in *NUP160*. None of these variants had been reported previously.

**Conclusion:**

This report indicates that biallelic variants in *NUP93*, *NUP107* and *NUP160* can cause severe early-onset SRNS, which rapidly progresses to ESKD. Moreover, these findings expand the spectrum of phenotypes and genotypes and highlight the importance of next-generation sequencing in elucidating the molecular basis of SRNS and allowing rational treatment for affected individuals.

## Introduction

Nephrotic syndrome (NS) is common in children. Most of them respond well to glucocorticoid, while a minority are steroid-resistant [1–3]. Steroid-resistant nephrotic syndrome (SRNS) primarily presents as focal segmental glomerulosclerosis (FSGS), which is associated with an unfavorable renal prognosis [4, 5]. Despite extensive research efforts, the etiology and pathogenesis of SRNS remain incompletely understood. Recent studies have identified an increasing number of genes associated with the development of SRNS, totally accounting for 29.5% of SRNS as reported by Hildebrandt [6]. About 66% of SRNS occurring within the first year of their life are caused by monogenic variants [7]. Thus far, more than 50 genes have been identified for SRNS worldwide [8]. Most of these gene products are located in slit diaphragm, cytoskeleton, mitochondria, lysosome and endocytic compartment of podocytes [8, 9].

Currently, variants in several nucleoporins (NUPs) have been identified as the underlying causes of SRNS. The nuclear pore complex includes a variety of NUPs that are distributed across the nuclear envelope and play a critical role in macro-molecular transportation between nucleus and cytoplasm [10, 11]. However, the reported cases of such variants are limited. Therefore, additional clinical and genetic data are required to characterize genotype–phenotype relationships and elucidate the role of NUPs in SRNS. This study aims to summarize the clinical and molecular genetic characteristics of four cases of SRNS caused by variants in *NUP93*, *NUP107* and *NUP160* genes with the intention of providing new insights into this rare disease.

## Patients and methods

### Case presentation

Case 1 was a 1-year-old female patient who initially presented with edema on the lower limbs and eyelids. She developed normally and was well-nourished. Upon physical examination, the patient presented with hypertension with a blood pressure of 115/68mmHg (≥95th percentile + 12 mmHg), while no other evident abnormalities were observed. The urine test showed proteinuria 3+ and hematuria 2+. The serum creatinine was 0.58mg/dl (eGFR 57ml/min/1.73m^2^). Subsequent renal biopsy revealed FSGS. Despite receiving treatment with a combination of prednisone and cyclosporin A, the patient remained nephrotic after approximately six months of treatment and eventually progressed to end-stage kidney disease (ESKD). Whole-exome sequencing (WES) identified biallelic variants in the *NUP93* gene.

Case 2 was a 5.3-year-old female patient who was hospitalized due to edema on lower limbs, with blood pressure of 141/108mmHg (≥95th percentile + 12 mmHg). Physical examination revealed no other significant abnormalities. Her growth and development were normal without any malformations. Urine examination showed hematuria 2+ and proteinuria 3+, while the serum creatinine level was 0.4mg/dl (eGFR 110ml/min/1.73m^2^). Despite four weeks of treatment with prednisone, the patient showed no response. So renal biopsy and whole-exome sequencing (WES) were recommended. The renal biopsy revealed global sclerosis in 11 glomeruli and segmental glomerulosclerosis in 4 among total 30 glomeruli. WES identified biallelic variants in the *NUP107* gene.

Case 3 involved a female child aged 10.5 years who presented with chest tightness and nausea. Physical examination showed hypertension with blood pressure ranging from 151/108 mmHg to 200/149 mmHg (≥95th percentile + 12 mmHg). There were no edema or growth and developmental delays. The plasma renin level was within the normal range, and the whole aortic computer tomography angiography (CTA) showed no remarkable findings. Echocardiography showed left ventricular hypertrophy with left ventricular ejection fractions (LVEF) at 38.7%. Ultrasound examination revealed no abnormalities in the kidneys, uterus, or ovaries. Urine analysis revealed a significant proteinuria level of 4+ without hematuria. The level of serum creatinine was 6.26 mg/dl (eGFR 9.6ml/min/1.73m^2^). Subsequently, the patient underwent peritoneal dialysis, and whole-exome sequencing (WES) identified biallelic variants in the *NUP107* gene.

Case 4 was a 3.5-year-old girl presenting with eyelid edema and hypertension. Urinalysis revealed proteinuria 4+ and hematuria 2+. The patient showed mild intellectual disability, as evidenced by a full-scale score of 48 on the Wechsler Preschool and Primary Scale of Intelligence (WPPSI), indicating an intellectual delay. A comprehensive neuropsychiatric assessment indicated delays in both gross and fine motor skills, adaptive abilities, language development and social behavior. Cognitive assessment demonstrated abnormal cognitive play and social communication behaviors. The Autism Behavior Checklist (ABC) confirmed the presence of autistic behaviors, with the Childhood Autism Rating Scale (CARS) indicating mild to moderate autism spectrum disorder. The ultrasound examination revealed the presence of a cord-like uterine, structure measuring 13 mm in length and 3 mm in anteroposterior diameter. The baseline level of serum creatinine was 0.34mg/dl (eGFR 114ml/min/1.73m^2^). However, there was an increase level in serum creatinine to 0.98mg/dl (eGFR 42ml/min/1.73m^2^) during the follow-up, suggesting the development of stage 3 chronic kidney disease (CKD3). Renal biopsy revealed FSGS. The patient was initially treated with prednisone and tacrolimus, which were discontinued due to no response. Thereafter WES identified biallelic variants in the *NUP160* gene.

**Fig. 1:**
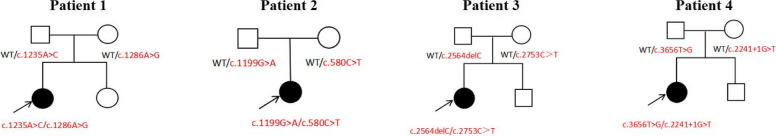
Family diagram of four patients. Black arrow indicates proband; WT: wild type

All of these cases had no family history of the disease. The specific family diagram is illustrated in Fig. 1.

### Detection and analysis of nucleoporin gene variants

After obtaining the informed consent of the patients’ parents, blood samples were collected from both the patients and their parents. DNA was extracted from peripheral white blood cells by using the MagPure Buffy Coat DNA Midi KF Kit according to manufacturer’s standard protocol. Genomic DNA was broken into 100–500 bp fragments by BGI’s enzyme kit (Segmentase, BGI), and 280–320 bp fragments were collected by magnetic bead. PCR amplification was performed using universal primers complementary to the adapter sequence to form a sequencing library. All amplified libraries were hybridized with exome capture probes (Agilent, USA) and sequenced. The clean reads derived from targeted sequencing and filtering were then aligned to the human genome reference (hg19) by using the BWA. Single-nucleotide variants (SNVs) and INDELs were detected with Sentieon (the same algorithm with GATK) analysis. The pathogenic variants were screened by ClinVar, OMIM, and HGMD databases. Functional prediction of missense mutations was conducted using PolyPhen-2, SIFT, and MutationTaster. All variants and potential pathogenic variants were validated via conventional Sanger sequencing methods.

## Results

### Clinical and renal pathological features and long-term outcome

**Fig. 2:**
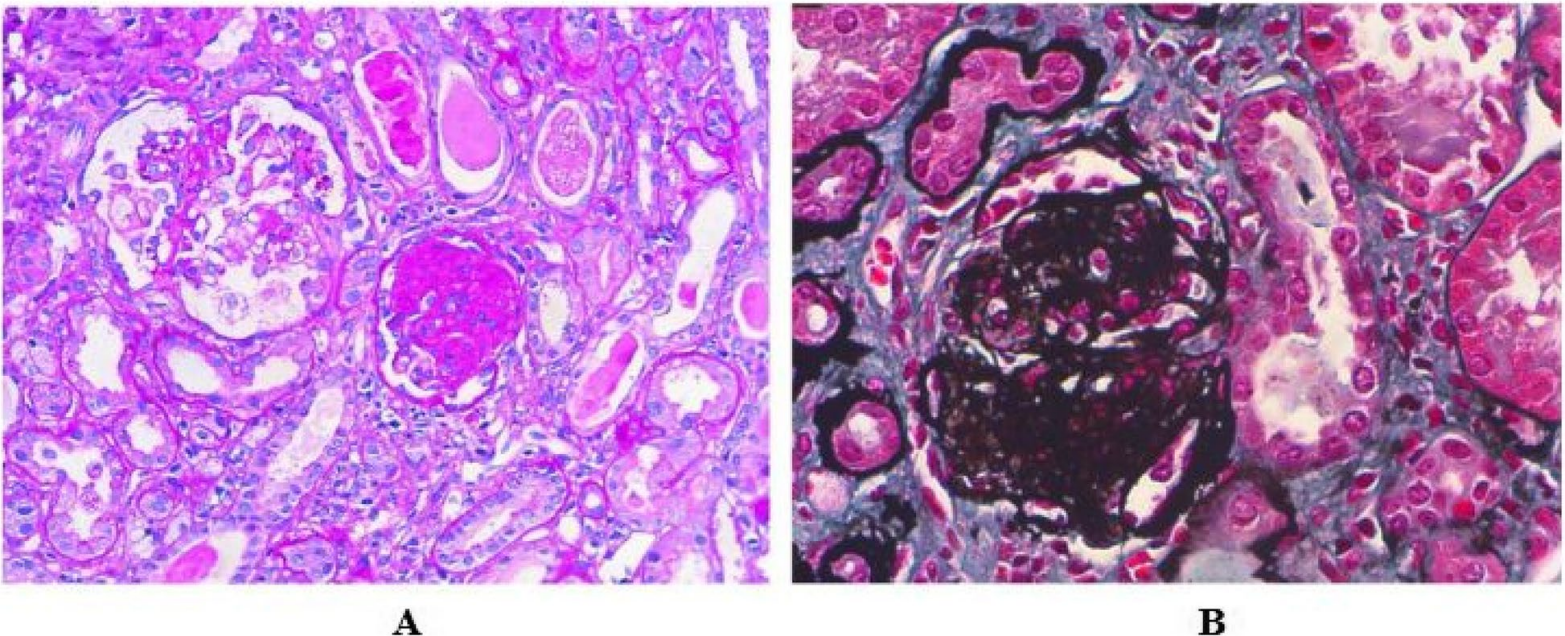
kidney biopsies reveal FSGS in the patients. A shows segmental sclerosis in a glomerulus and a global sclerosis in another glomerulus (HE, 100). B indicates global glomerulosclerosis (PASM, 100)

The clinical and laboratory data of four patients are shown in Table 1. All patients initially presented with massive proteinuria with or without hematuria. Patient 2 and 4 had normal serum creatinine levels and eGFR at the onset, while patient 1 and 3 had elevated levels of serum creatinine and impaired renal function at beginning. Moreover, patient 3 presented with heart failure as an extra-renal manifestation, and patient 4 showed mild intellectual disability and uterine dysplasia. Renal biopsy revealed focal and segmental glomerular sclerosis (Fig. 2).

**Table 1:** The clinical and renal pathological features of 4 patients at diagnosis

	Patient 1	Patient 2	Patient 3	Patient 4
Age at onset (years)	1	5.3	10.5	3.5
Age at diagnosis (years)	1	5.3	10.5	3.5
Gender	Female	Female	Female	Female
Renal manifestation				
Edema	Y	Y	N	Y
Hypertension	Y	Y	Y	Y
oliguria	Y	Y	Y	Y
Extra-renal manifestation	N	N	HF	UD, ID
Renal pathology	FSGS	FSGS	NA	FSGS
Hematuria	2+	2+	-	2+
Proteinuria	3+	3+	4+	4+
24hUP (mg/24h)	4000	2245.6	NA	2828.3
Serum albumin(g/L)	20.9	28.6	31.6	20
Serum creatinine at onset (mg/dl)	0.58	0.4	6.26	0.34
eGFR at onset (ml/min/1.73m^2^)	57	110	9.6	114
eGFR at last follow-up (ml/min/1.73m^2^)	9.0	5.0	9.6	42
Age of ESKD (years)	1.5	9	10.5	NA

*Y* Yes, *N* No, *HF* Heart failure, *UD* Uterine dysplasia, *ID* Intellectual disability, *FSGS* focal segmental glomerulosclerosis; *NA* Not applicable; *24hUP* 24 hour urine protein

Patient 1 rapidly progressed to ESKD within six months of onset and regrettably passed away at 1.5 years of age. Patient 2 developed renal failure at 9 years old and subsequently underwent renal transplantation. Patient 3 was diagnosed with ESKD at the initial presentation and was on maintenance peritoneal dialysis. And patient 4 was diagnosed with CKD3 at 5 years old during the follow-up period (Table 1).

### Identification of pathological variants in the *NUP* genes

Biallelic variants in the *NUP* genes were detected by WES in all patients. Specifically, patient 1 had biallelic missense mutations c.1235A>C (p.Tyr412Ser) and c.1286A>G (p.Tyr429Cys) in the *NUP93* gene. Patient 2 showed biallelic variants c.1199G>A (p.Gly400Glu) and c.580C>T (p.Arg194*) in the *NUP107* gene, and patient 3 also had biallelic variants c.2564delC (p.Pro855fsTer*23) and c.2753C>T (p.Pro918Leu) in *NUP107*, resulting in amino acid substitutions and proteins truncation. Patient 4 was identified with a missense mutation c.3656T>G (p.Leu1219Trp) and a splicing donor mutation c.2241+1G>T in the *NUP160* gene. Ployphen2, MutationTaster analysis indicated that all the missense mutations were harmful (Table 2).

**Table 2:** Gene variants and pathogenic analysis in 4 patients with *NUP* variants

	Gene	Mutation	From	Amino acid	Ployphen2	Mutation Taster	ACMG
Patient 1	NUP93	c.1235A>C	Father	p.Tyr412Ser	1.00	D	PM2+PP2+PP3
		c.1286A>G	Mother	p.Tyr429Cys	0.655	D	PM2+PP2+PP3
Patient 2	NUP107	c.1199G>A	Father	p.Gly400Glu	1.00	D	PM2+PP2+PP3
		c.580C>T	Mother	p.Arg194*	NA	D	PVS1+PM1+PM2
Patient 3	NUP107	c.2564delC	Father	p.Pro855fsTer*23	NA	D	PVS1+PM1+PM2
		c.2753C>T	Mother	p.Pro918Leu	1.00	D	PM2+PM3+PP3
Patient 4	NUP160	c.3656T>G	Father	p.Leu1219Trp	1.00	N	PM2+PM3+PP3
		c.2241+1G>T	Mother	NA	NA	D	PVS1+PM2

*NA* Not applicable, *D* Disease causing, *N* Polymorphism

### Clinical and molecular genetic characterization of reported patients carrying biallelic variants of *NUP* genes

**Fig. 3:**
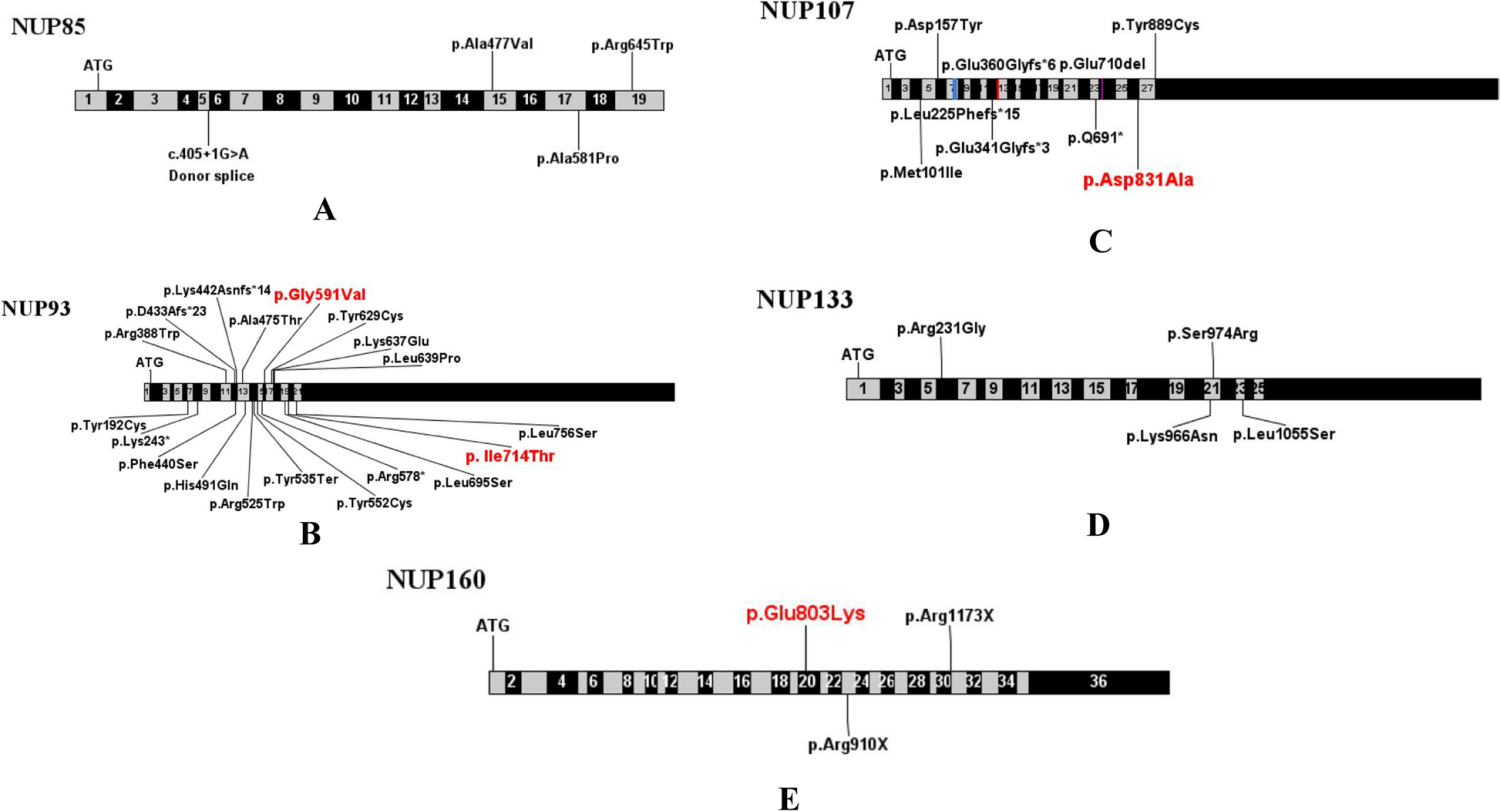
Schematic illustration of mutation sites in genes of *NUP85* (**A**), *NUP93* (**B**), *NUP107* (**C**) , *NUP133* (**D**) and *NUP160* (**E**). Hot spot mutations are indicated in red

To date, a total of 60 cases of *NUP*-associated SRNS have been reported worldwide (Table 3). Among these reported cases, variants of *NUP93* gene were observed in 24 individuals (40%), while *NUP107* variants in 20 individuals (33.3%). Additionally, there were also eight cases (13.3%) with *NUP133* mutations, four cases (6.7%) with *NUP85* mutations, one case (1.7%) with *NUP205* mutation and three cases (5%) with *NUP160* mutations. The majority of these reported cases initially presented with SRNS and eventually progressed to ESKD. The detailed information of these variants were presented in Table 3 and Fig. 3, involving 22 variants in *NUP93*, 11 in *NUP107*, 6 in *NUP133*, 4 in *NUP85*, 1 in *NUP205*, and 3 in *NUP160*. Missense mutations were the majority of all *NUP *mutations.

**Table 3:** Summary of clinical and genetic features of reported patients with *NUP* variants

Gene	F/M	No of cases	Age of onset (years) (Min-Max)	Clinical manifestations	Renal biospy (%)	ESRD at last follow-up (n/%)	Age at ESRD (years) (Min-Max)	Extra renal	Variant
*NUP93* [12–21]	12/12	24	3.95 (0.58-9)	SRNS	FSGS(75%), MCD(4.2%), IFTA(8.3%), NA(12.5%)	22/91.7%	5.0 (0.83-11)	ASD, HF, MGS, erythrocytosis	c.575A>G, c.727A>T, c.1162C>T, c.1298delA, c.1319T>C, c.1326delG, c.1423G> A, c.1473T > G, c.1537+1G>A, c.1538-6A > G, c.1573C>T, c.1605C>G, c.1655A>G, c.1732C>T, c.1772G>T, c.1886A>G, c.1909A>G, c.1916T>C, c.2084T > C, c.2137-18G>A, c.2141T>C, c.2267T > C
*NUP107* [22–25]	10/10	20	3 (2-14)	SRNS	FSGS(80%), DMS(5%), MCD(5%), NA(10%)	17/85%	6.79 (4-13)	MC, ID, SS, DD, FD, DCM, HTN	c.303G>A, c.469G>T, c.627_663dup37, c.969+1G>A, c.1021dupG, c.1079_1083del, c.1735-3T>G, c.2071C>T, c.2129_2131delAAG, c.2492A>C, c.2666A>G
*NUP160* [25, 26]	2/1	3	7 (7-16)	SRNS	FSGS(66.7%), NA(33.3%)	1/25%	15	UD, ID	c.2407G>A, c.2728C>T, c.3517C>T
*NUP85* [25]	2/2	4	7.5 (4-11)	SRNS	FSGS(75%), NA(25%)	3/75%	10 (7-12)	SS, ID, GHD	c.405+1G>A, c.1430C>T, c.1741G>C, c.1933C>T
*NUP133* [25, 27, 28]	NA	8	3.37 (0.92-10)	proteinuria	FSGS(25%), NA(75%)	8/100%	6 (1.83-20)	ID, MC, GD, HI, Epilepsy, GAMOS	c.182+387T>G, c.691C>G, c.2898G>C, c.2922T>G, c.3164T>C, c.3335-11T>A.
*NUP205* [16]	1/0	1	3	SRNS	FSGS(100%)	1/100%	7	No	c.5984T>C

*MCD* Minimal change disease, *IFTA* Interstitial nephritis with tubular atrophy, *NA* Not applicable, *ASD* Autism spectrum disorder, *HF* Heart failure, *MGS* Marcus-Gunn-syndrome, *DMS* Diffuse mesangial sclerosis, *MC* Microcephaly, *ID* intellectual disability, *SS* Short stature, *DD* Developmental delay, *FD* Facial dysmorphism, *DCM* dilated cardiomyopathy, *HTN* Hypertension, *UD* Uterine dysplasia, *GHD* Growth hormone deficiency, *GD* Growth deficiency, *HI* Hearing impairment, *GAMOS* Galloway-Mowat syndrome

## Discussion

NUPs are situated in the nuclear membrane, and approximately 30 kinds of NUPs assemble to form the nuclear pore complex (NPC), serving as a special and unique transport channel across the nuclear membrane [29]. The NPC comprises a core scaffold, a nuclear basket, transmembrane nucleoporins and a central selective channel [11]. NUP93 is located in the inner ring of the core scaffold, while NUP107 and NUP160 are located in the outer ring also known as the “Y” complex [30]. NUP93, NUP107 and NUP160 interact with other NUPs and participate in the assembly of NPCs, which are crucial for trans-nuclear membrane transportation. Any alterations in the NUPs or defects in transport channels can hinder transmembrane transport, resulting in the abnormal accumulation of materials in nucleus or cytoplasm. Consequently, variants in NUPs are associated with a variety of diseases [31]. Previous studies have linked *NUP93*, *NUP107* and *NUP160* to cancer, congenital heart disease, neurological diseases and gonadal dysgenesis [32–36]. Recently several SRNS cases caused by variants in *NUPs* have been reported and attracted the attention of pediatric nephrologists.

Previous studies had indicated that variants in *NUP93* and *NUP107* were the most frequent mutated *NUP* genes in individuals with SRNS. Patients with variants in these genes rapidly progressed to ESKD at a young age. Conversely, cases of SRNS with *NUP160* variants presented relatively later and progressed to ESKD at an older age than those with *NUP93* and *NUP107* variants. Furthermore, patients with variants in *NUP93*, *NUP107*, and *NUP133* were more likely to present with extra-renal manifestations.

Previously reported *NUP* variants included missense, nonsense, frameshift, small deletion and splicing mutations, among which missense mutations were found to be the most prevalent. Notably, variant hotspots were found in *NUP93* and *NUP107* genes. Specifically, the c.1772G>T (p.G591V) variant in *NUP93* was identified as a pathogenic European founder variant and the c.1537+ 1G>A (deletion of exon 13) was found to be another pathogenic variant in Germans [13]. The c.2492A > C (D831A) variant in the *NUP107* gene was considered to be unique to East Asians [23]. Furthermore, the variant c.2407G>A (p.Glu803Lys) in the *NUP160* may be a hotspot variant in Asian according to previous reports.

The specific mechanisms underlying steroid-resistant nephrotic syndrome (SRNS) caused by *NUPs* remain unclear. A previous study demonstrated the presence of glomerular dysplasia and abnormal podocyte processes in zebrafish models with *NUP107* knockdown [22]. Knockdown of *NUP160* gene resulted in podocyte proliferation incapability, increased apoptosis, autophagy and cell migration, and altered expression and localization of nephrin, podocin, CD2AP and α-actinin-4 [37]. Furthermore, Braun [25] discovered upregulation of cdc42 in podocytes with knockout of *NUP85*, *NUP107*, and *NUP133* genes. In addition, knockdown of *NUP93* gene in podocytes disrupted BMP7-dependent SMAD signaling, potentially implicating it in the pathogenesis of SRNS [16].

Next-generation sequencing (NGS) is a powerful and high-throughput genetic test helping to identify the causative variants of genetic diseases. This technique offers valuable insights and evidence for the purposes of diagnosis, treatment, and genetic counseling [38–40]. Targeted panel sequencing is faster and cheaper than WES, making it a preferred choice for patients whose clinical data are highly consistent with a specific genetic defect or a known group of genes [41]. However, the limitation of targeted panel is that only genes within the panel are sequenced, which may result in missing genes located beyond the panel. As the cost of sequencing becomes cheaper and cheaper, WES has been widely accepted especially for patients with ambiguous phenotypes that make it difficult to apply targeted panel sequencing [42].

Cystic kidney diseases and renal tubulopathies subjected to WES result in relatively high rates of positive findings [43]. Generally speaking, an earlier onset of a disease is associated with a higher possibility of genetic etiology. Identifying the causes of sporadic non-syndromic SRNS is challenging. Overall, WES should be considered for SRNS patients with multisystem involvement as well as with unexplained clinical manifestations. In this study, all four patients lacked specific manifestation and two of them showed multisystemic involvement. Therefore, gene variant screening was the only way to identify genetic causes. Fortunately WES uncovered definitive pathogenic biallelic variants of *NUP* genes, thereby the ineffective treatment of prednisone and immunosuppressants was immediately discontinued. Our report demonstrates the necessity and diagnostic utility of genetic analysis in sporadic cases of SRNS and highlights the role of NGS in understanding the molecular mechanisms of SRNS and facilitating rational and individualized treatment for patients.

This paper is believed to be the first to report on a series of Chinese cases of SRNS associated with variants of *NUP* genes. Furthermore, all these variants in *NUP93*, *NUP107* and *NUP160* have not been previously reported. Since this study is solely a case report, the limited number of cases prevented us from drawing conclusive phenotypic and genotypic correlations. In addition, how *NUP* variants cause SRNS remains unclear and requires further comprehensive molecular research.

## Conclusion

In summary, this study reports four cases of sporadic SRNS caused by novel variants in the *NUP93*, *NUP107*, and *NUP160* genes in Chinese children. The findings extend the spectrum of phenotypes and genotypes, and also highlight the importance of NGS in elucidating the molecular mechanisms of SRNS and allowing personalized treatment for affected individuals.

## Data Availability

The datasets used and/or analysed during the current study are available from the corresponding author on reasonable request.

## Abbreviations

SRNS: Steroid-resistant nephrotic syndrome
ESKD: End stage kidney disease
CKD: Chronic kidney disease
CKD3: Stage 3 chronic kidney disease
NUP: Nucleoporin
WES: Whole-exome sequencing
NPC: Nuclear pore complex
NGS: Next-generation sequencing
